# Pathogenesis of HSV and CMV Infections in Pregnancy

**DOI:** 10.1155/S1064744997000215

**Published:** 1997

**Authors:** Myriam Askienazy-Elbhar, Christophe Sifer

**Affiliations:** 1 Laboratoire de Biologie Magenta 41 Boulevard de Magenta Paris 75010 France; 2 Laboratoire de Virologie Groupe Hospitlier Pitié-Salpétrière 83 Boulevard de l'Hôpital Paris Cedex 13 75651 France

## Abstract

Human herpesvirus (HHSV) and human cytomegalovirus (HCMV) infections during pregnancy are a major concern of public health because of the risk for severe sequelae for the fetuses and the neonates and because primary infections, reinfections and reactivations can be asymptomatic. The risk for neonatal herpes is mostly congenital, while the risk for HCMV infection is either prenatal or congenital. Screening exposed women has not brought definite solutions but is currently being evaluated. Among pregnant women with active infection, evaluation of the fetus for contamination and thus for the risk for severe immediate or long-term sequelae for neonates is the major goal. Diagnostic tools are available, cell culture still being the gold standard, and polymerase chain reaction (PCR) being currently evaluated for its contribution to diagnosis of active infection. Consensus for screening pregnant women as well as achievement of antiviral vaccines are the most urgent intervention strategies to develop in the near future.


Infectious Diseases in Obstetrics and Gynecology 5:133–141 (1997)
© 1997 Wiley-Liss, Inc.

### Pathogenesis of HSV and CMV Infections in Pregnancy

**Myriam Askienazy-Elbhar and Christophe Sifer**
*Laboratoire de Biologie Magenta, 41 Boulevard de Magenta, 75010 Paris (M. A.-E.); Laboratoire de Virologie, Groupe Hospitlier Pitié-Salpétrière, 83 Boulevard de l'Hôpital, 75651 Paris Cedex 13 (C.S.), France*

ABSTRACT

**Human herpesvirus (HHSV) and human cytomegalovirus (HCMV) infections during pregnancy are a major concern of public health because of the risk for severe sequelae for the fetuses and the neonates and because primary infections, reinfections and reactivations can be asymptomatic. The risk for neonatal herpes is mostly congenital, while the risk for HCMV infection is either prenatal or congenital. Screening exposed women has not brought definite solutions but is currently being evaluated. Among pregnant women with active infection, evaluation of the fetus for contamination and thus for the risk for severe immediate or long-term sequelae for neonates is the major goal. Diagnostic tools are available, cell culture still being the gold standard, and polymerase chain reaction (PCR) being currently evaluated for its contribution to diagnosis of active infection. Consensus for screening pregnant women as well as achievement of antiviral vaccines are the most urgent intervention strategies to develop in the near future. Infect. Dis. Obstet. Gynecol. 5:133–141, 1997.** © 1997 Wiley-Liss, Inc.

KEY WORDS
Primary infection, reactivation, reinfection, neonatal herpes, cytomegalic inclusion disease, cytopathic effect, latency, persistence, immunocompetency, screening

HHSV and HCMV, members of the Herpesviridae family, are causal agents of ubiquitous infections. Although mostly asymptomatic, their common feature is to dangerously affect childbirth if infection occurs during pregnancy or delivery. For both viruses, primary infection during pregnancy is a major public health issue because of severe risks for the child. After primary infection the viruses never leave the host and enter into a latency or persistence state which is the key to the immunopathogenesis of the infection.

Prevention and treatments are available, but for both infections, screening exposed women is controversial because the risk for neonatal contamination is not clearly estimated.

We will revew the pathogenesis of both viruses for pregnant women and expose intervention strategies. Herpesviridae family, composed of more than 100 members, is divided in 3 subfamilies, α-, β- and γ-herpesvirinae, according to their biological criteria, like cycle duration, latency or persistence in the host, and their oncogenic properties. Eight herpesviruses infect the human. Herpes-simplex virus type 1 (HSV1) and type 2 (HSV2) are responsible for labial and genital herpes, respectively, and varicella and zona virus (VZV) are α-Herpesvirinae. Human cytomegalovirus (HCMV), HHV type 6 and HHV type 7 are β-herpes virinae, marked by their opportunistic pathogenesis. Epstein-Barr virus (EBV) agent of the infectious mononucleosis, linked to Burkitt lymphoma and nasopharynx carcinoma, and HSV type 8 found in Kaposi disease lesions, are γ-herpesvirinae marked by their oncogenic properties.

Address reprint requests to: Myriam Askienazy-Elbhar, Laboratoire de Biologie Magenta, 41 Boulevard de Magenta, 75010 Paris, France

Received 1 October 1997

Accepted 21 October 1997

### GENERAL PROPERTIES OF HSV AND CMV

These two viruses share the general morphology of the herpesviruses. Their double stranded linear DNA is surrounded by an icosaedric capside forming the nucleocapside; enclosed in an envelope formed by a double layer of cellular phospholipids and viral glycoproteins, are the nucleocapside and the tegument, amorphous material containing proteins without DNA. Host cell penetration by the two viruses occurs through still unknown cellular receptors. The virus binds by glycoprotein C (gC) or secondarily by glycoprotein B (gB)[1] to cellular heparin sulfate type proteoglycans.[2] The viral replication takes place in 3 phases.[3]

The very early phase corresponds to the immediate early proteins (IE) synthesis, transactivated by the tegument proteins.[4] The early phase, where the early (E) proteins are synthetised after transactivation by the IE proteins, opens to DNA synthesis. Then structural protein synthesis corresponds to the late phase.

HHSV and HCMV genomes are constituted of a linear double stranded DNA of 155 Kbp and 240 Kbp respectively,[5] organized in two single units, a long one (UL) and a short one (Us) framed by repeated reverse sequences (TRL and IRL for UL, IRS and TRS for Us).[6] The differential orientation of those single units allows 4 isomeres for each genome.[7] These genomes contain 100 opening reading frame (ORF) for HHSV and more than 200 ORF for HCMV. HSV1 and HSV2 share more than 50% of nucleotidic sequence homology.[8]

The leader of the subfamily of α Herpesviridae, HHSV1 is characterized by a broad host spectrum in vitro, narrow in vivo, a short viral cycle, latency and reactivation properties, a viral thymidine kinase mediated neurovirulence[9] and a gD and gB mediated neuroinvasion.[10]

The leader of the subfamily of β Herpesviridae, HCMV is characterized by a narrow host spectrum in vitro, broad in vivo, a long viral cycle (abortive = dense corpuscles synthesis or permissive = virion synthesis), and properties of persistence and reactivation.

### PATHOGENESIS

### Epidemiology

Humans are the only natural reservoir of HHSV and HCMV.

Infection seroprevalence in adults is 70% for HHSV1 and 10 to 60% for HHSV2[11] depending on socioeconomic status and sexual habits.

Contamination occurs mostly by contact with oropharyngeal or genital secretions. HCMV seroprevalence varies with socioeconomic status, age and sex (women are more often infected than men). In France, the HCMV seropositivity rate is around 50% by the age of 35.[12,13,14,15] The rate peaks at 80% at the age of 3 in developing countries.[16,17,18] Transmission can occur by blood, tears, maternal milk, semen, genital secretions, urine, saliva, and by transplacental passage during the viremia which accompanies the active infection, either primary infection (p.i.), reinfection or reactivation.

### Clinical Features

HHSV1-2 p.i. is most of the time not apparent. HHSV1 p.i. symptoms are variable. Gingivostomatitis with dysphagia, fever and cervical adenoïds, or kerato-conjunctivitis, or encephalitis, can be found. HHSV1 can also infect the genital tract. HHSV2 p.i. can be responsible for genital vesicular lesions with fever and inguinal adenoids, and for neonatal herpes (NNH). Rare cases of fulminating hepatitis have been described in pregnant women during p.i. Clinical forms of reactivation are labial herpes for HHSV1 and genital herpes or neonatal herpes for HHSV2. Those reactivation forms are localized or disseminated according to the immunocompetency or the immunodepression of the host.

HCMV p.i. may not be apparent. The virus is responsible for 8% of the mononucleotic syndroms[19] and for a condition of which importance is directly correlated to the level and type of the host immunodepression: HCMV is the infectious agent responsible for opportunistic infections of viral prognosis, either with the p.i. (60% incidence in the transplanted) or with reinfections (40%) or reactivations (20%). Moreover, the frequency of those pathologies varies with the type of immunodepression. 20% of bone marrow transplants develop a very severe interstitial pneumopathy (80% of all HCMV diseases in this population) versus hardly 1% of better prognosis, in the AIDS population. Conversely, 10 to 15% of HIV seropositives at AIDS phase with CD4+<100/mm3 develop a retinitis (80% of all HCMV diseases in this population) and more than 5% an ulcerative colitis, those two conditions being very rare in the bone marrow transplant population. HCMV is also responsible for encephalitis, hepatitis, leucopenia in those immunodepressed populations, as well as congenital infection of variable severeness. Some Guillain-Barré syndromes have been described, especially in pregnant women.

### Latency

The site of HHSV latency has been located by in situ hybridization (ISH). The viral genome has been found in neuronal cells of neurosensitive adenoids.[20] Molecular studies have demonstrated the existence of latency associated transcripts (LAT), viral transcription products present only during latency.[21] This anatomically well defined latency explains the clinical identity of recurrent episodes caused by an acute stress.

The location of HCMV latency is still unknown, and absolute evidence of latency has not been brought yet for this virus since no cellular line has allowed to obtain the virus in latency without replication.

Nevertheless we know that it persists for the lifetime of the host.[22] ISH assays have shown its presence in monocytes, epithelial and endothelial cells of different organs of healthy individuals.[23] Virus and host maintain a balance status in such a way that the cell cannot get rid of the virus but prevents virus replication. Animal models have shown that replication and dissemination rate during primary infection determine the probability of latency in a given organ, and that those factors are more important in neonatal than in adult infection.[24] It is likely that HCMV DNA is not integrated in the cell genome and that the virus replication is regulated by cellular transcription factors.

HCMV is coated with β2 microglobulin chains which hide the epitopes recognized by neutralizing antibodies. There is a low rate of replication rather than a latent infection for this virus.

HCMV reactivation occurs after an immunodepressive state, either iatrogenic (graft, chemotherapy) or natural (AIDS, pregnancy, stress), and the associated viremia explains the clinical features of this reactivation.

### IMMUNOPATHOLOGY

### *HHSV1-2*

Antibodies are responsible for protection from reinfection and for neutralisation of the neuroinvasion in adults.[25] That is why herpetic encephalitis is not more frequent in immunodepressed or in immunocompromised patients. However, the transplacentae transfer of humoral immunity does not always protect the newborn from perinatal infection. CD4+ lymphocytes are responsible of virus elimination at the periphery.[26] Thus, HHSV1-2 infections are easily disseminated in immunodepressed patients. Moreover, their ocular autoreactivity gives its immunopathogenetic component to the herpetic keratoconjunctivitis. They have no role in herpetic encephalitis, where the demyelinating inflammatory reaction is associated with the virus multiplication. CD8+ lymphocytes control the infection.

The neonate immune status is quite important when congenital infection is concerned. There is a relation between the maternal antibody transmission rate to the fetus and the development of NNH. This factor, associated with a decreased T cell response in the newborn, exposes the fetus to NNH infection.[27]

### *HCMV*

Virus replication occurs only in differentiated cells and is responsible for an increase of the global cellular metabolism. HCMV is responsible for nonprotecting antibody synthesis, which does not prevent especially reinfection by another virus strain. The infected cell decreases the synthesis of certain antigens of the major histocompatibility complex (MHC) class II, partly protecting itself from the immune reaction of the host. An UL18 HCMV gene, homologous to MHC class I molecules, can efficiently inhibit infected cells lysis by natural killer (NK) cells.[28] Whether these homologues are really expressed on the infected cell surface and how they affect NK lysis must still be clarified. Controlling HCMV infection depends on a specific response of the cell mediated immune system.[29] Most of the MHC restricted cytotoxic cells specifically recognize one single structural protein, phosphoprotein 65 (pp 65). The healing of a HCMV infection is accompanied by an increase of CD8+ lymphocytes and NK cells. Healthy patients seropositive for HCMV and seronegative patients demonstrate an NK activity specific for the HCMV infected cells, but this activity is stronger in HCMV seropositives, which suggests its role in the virus-host balance during viral persistence. A good NK

response is positively associated with the lack of symptoms in the congenitally infected child and with the favorable evolution of the HCMV disease in bone marrow transplants. HCMV immunopathogenesis is asserted by virus replication at the site of clinical manifestations, but also by cellular destruction, and modification of cellular functions, especially cytokine production. Moreover, HCMV is known to be immunodepressive: during the acute phase of the HCMV mononucleotic syndrome, there occurs susceptibility to secondary infections, reversal of the CD4+ (helper)/CD8+ (supressive) ratio, and autoantibody induction. In vitro lymphoproliferation with specific or non specific HCMV antigens, cytotoxic cell mediated activity, and peripheral blood leucocytes NK activity, are decreased,[30] while differentiation cytokine and growth factor production are stimulated. In fact, interrelations between this virus and cytokine production by infected cells modifying the immune status of the considered tissue are extremely complex, especially because the modifications vary with the type of the infected cell.[31,32]

### NEONATAL HERPES

The frequency of NNH is 1 to 5/10000 births with 1 HSV1 to 4 HSV2 cases. The disease is unexpected in two thirds of cases.[33] Transmission occurs by genital contact during delivery. The absence of viremia in immunocompetent individuals excludes fetal contamination through the placenta. However, in utero infections are reported, mostly mediated by placentitis.

The mother exhibits no signs nor symptoms in two thirds of cases.

In maternal genital p.i. at delivery, the risk for NNH is 75% (no antibodies, virus +++). In maternal genital recurrence at delivery, NNH risk is 3 to 5% (antibodies+/virus+).[34] If there is a maternal or paternal genital history, the risk is 10%. As for any female patient, the risk for NNH is 1% (0.1 to 1% are asymptomatic excretors).[35] The diagnosis is made by sampling the vesicles at the infected sites and culturing them on human fibroblasts. The cytopathic effect (CPE) is read within 48 hours. Cytodiagnostic tests and ELISA can also be used.

In case of a previous history of genital herpes, the diagnosis of an asymptomatic excretion at the cervix is made by cell culture or direct immunofluorescence assay (DIFA). Gene amplification with PCR is currently being evaluated for this indication.[36] This very sensitive technique is of controversial benefit because it does not differentiate latency from active infection, which is essential for NNH risk evaluation.

For the child, asymptomatic forms are exceptional. There are severe disseminated forms (icteria, purpura, hepatosplenomegaly, respiratory signs associated or not associated with vesiculous lesions), severe forms localized to the central nervous system (CNS) resulting in encephalitis. Those two forms are of redoubtable prognosis.

Benign forms, localized in skin or mouth, are of better prognosis.[37] The embryopathy resulting from early maternal fetal transmission is exceptional. NNH mortality or irreversible sequelae are 50% without treatment.[38]

A quick diagnosis of vesicular lesions can be made by DIFA, ELISA or electron microscopy. Virus can be isolated by cell culture of vesicles, tears, blood, pharyngal samples, urine, and, less constantly, cerebrospinal fluid, blood and biopsies.[39]

Treatment as a pediatric emergency should be started as soon as NNH is clinically suspected. Intraveinous acyclovir (ACV), 10–15 mg/kg 3 times a day for 10 to 14 days is the elective treatment for NNH, thanks to which two thirds of the infected children live without sequelae.

The primary prevention of NNH to avoid prepartum primary infection is sexual education of the future parents (monogamy, condom use). Labial herpes bearers should refrain from kissing neonates.[40]

Three strategies are available in the prepartum stage: cesarean section, iodine aseptization of the genital tract, and ACV administration to the mother and/or child. The choice of the strategy depends on the patient's situation towards genital herpes.

Four situations can be found (Table 1):

Situation 1 is a primary infection prepartum or in the previous month: cesarean section is recommanded in the month before delivery, if possible, with ACV administration to the neonate, especially if the cesarean section has been delayed or performed after rupture of membranes. The mother can also be treated by ACV if she presents severe clinical symptoms with maternal risk.

Situation 2 is recurrent herpes in the prepartum or in the preceeding days: in this case, only the cesarean section is recommended in the week be-

TABLE 1. The maternal genital herpes manifestations in relation to neonatal herpes

| Maternal situation | Frequency in infected children's mothers | Neonatal herpes risk | Recommended proposition |
|---|---|---|---|
| Prepartum primary infection (or in the preceeding month) | Rare | ++++ = 75% | Cesarean section Discuss ACV |
| Recurrence in prepartum or in the preceeding days | + | ++ = 2 to 5% | Cesarean section |
| Maternal or paternal history of genital herpes only | ++ | + = 1/1000 | Vaginal delivery after vaginal swab and iodine aseptization, if positive, discuss ACV |
| No herpes history no clinical manifestation | ++++ 2/3 cases | +/– = 1/1000 | No intervention except avoid any STD |

0.1 to 1% of all pregnant women have an asymptomatic genital excretion of HHSV.

fore the expected term. An alternative prevention strategy could be a long term ACV oral treatment of pregnant women suffering frequent HHSV2 recurrent episodes.[41]

Situation 3 is a maternal or paternal history of genital herpes. In this case, vaginal delivery is advised after genital iodine aseptization. Previously, asymptomatic excretion of HHSV will be looked for, and if positive, ACV will be given to the neonate. In every situation with a risk for NNH, if monitoring is used, electrodes must not be transfered from the mother to the newborn to avoid herpetic encephalitis.

In situation 4 where no herpes history nor clinical manifestation is known, the only prevention is to avoid any sexually transmitted infection during pregnancy by primary prevention strategies.

### HCMV CONGENITAL AND NEONATAL INFECTION

HCMV is the primary cause of congenital viral infections in the world, occuring in 0.4 to 2.3% of neonates,[14a,14b] of whom only 5–10% are symptomatic. Of the 90% asymptomatic cases, 10% will have an abnormal development.[42] But infection and HCMV disease must be carefully differentiated.

In contrast to what happens with HHSV, clinical manifestations are exceptional, corresponding for 1/30 to CID (cytomegalic inclusions disease), the most severe case of HCMV congenital infections.[43] CID frequency is about 0.03%. Contamination occurs through ascension from urogenital secretions or mother's viremia, into the placenta by blood leukocytes.[44] The virus reaches the fetus through the placenta barrier and replicates in elected organs, mostly kidneys,[45,46,47] and is excreted in the amniotic fluid.

The most severe cases result from a maternal p.i. during pregnancy. Transmission rate to the fetus varies from 30 to 50%.[42,48,49,50] The risk for p.i. during pregnancy varies from 1 to 13% with the studies and the countries.[51,52,53] and increases with the number of pregnancies (2.3% at first pregnancy vs. 3.8% at the second).[49] The presence of endometritis or ovaritis implies the possible fetal infection by viral ascension or proximity. Asymptomatic neonatal HCMV infections relate to viral reinfections and particularly reactivations during the third trimester of pregnancy.More than 10% of pregnant HCMV seropositive women excrete the virus through uterine cervix during that period.

During secondary infections, after reactivation, persistence of chronic infection or reinfection by an other virus strain,[54,55] the maternal fetal transmission rate varies from 0.5% to 10%.[48,49,56] The infants are not protected from an acute infection by maternal antibodies but from severe CNS disease. Perinatal HCMV infection is very frequent. In a population where 1% of children are viruric at birth, 12 to 15% of the neonates are viruric at 1 month of age, due to the transmission by the maternal milk[57,58] or any other intimate contact. It is asymptomatic in most cases, with 5 to 15% of infraclinic in utero-aquired encephalitis leading to mild neurotic sequelae or deafness.[59,60a,60b]

In the mother, alarm signs are exceptionally present in rare cases of maternal p.i. with fever, mononucleotic syndrom, arthralgia, and moderate raise of hepatic ALAT. This p.i. very rarely can result from a blood transfusion from a seropositive donor to a seronegative receiver. It may occur that attention is drawn at ultrasound examination to an important fetal growth retardation, especially with intraskull calcifications.

In that case, p.i. diagnosis would be easily confirmed by virus detection in blood or urine by culture on human fibroblasts. Evidence of viral IE proteins by immunocytochemistry allows to estimate the viral titer in 48 hours. This viremia or viruria asserts the active infection without distinction of p.i. or secondary infection.

Determination of HCMV antigenemia in the nuclei of circulating polymorphonuclear leukocytes after cytocentrifugation by a monoclonal antibody to pp65[61] gives in 3 hours an answer with a good sensitivity compared to cell culture. This semiquantitative antigenemia is of real diagnostic interest in AIDS-associated HCMV diseases, but has not yet been evaluated in pregnant women.

Circulating blood leukocytes PCR is currently being evaluated for its contribution to the diagnosis of active infection in pregnant women; but the problem is the same as it is for HHSV1-2. Preliminary studies currently qualify and quantify HCMV genome detection sites and detection proteins, and try to correlate the active infection rate to resulting clinical symptoms (hybrid capture, RT-PCR, serum-PCR).

Indirect diagnosis of HCMV infections is difficult. In the presence of alarm signs, p.i. would be easily confirmed by a seroconversion, which requires two blood samples.

However, the high prevalence of HCMV in the general population is an obstacle to the diffusion of a serologic test for pregnant women. A single serum can only tell if the patient is seropositive or negative at the time of the test. AntiCMV IgM is lacking in 11 to 30% of primary infections and thus is not a certainty test for p.i., and when it is present, it is not absolute evidence of active infection since it can last for a year after primary infection, and 5 to 10% of secondary infections produce IgM. Laboratories have the legal obligation to keep sera frozen at −20°C for one year. This could allow the differentiation of a p.i. from a reactivation in the presence of IgM if there were no IgM in the first serum. Moreover, given the interactions between herpetic infections and nonspecific reactivations of the immune system (like antiCMV $\beta$ clones nonspecific reactivation), serologic variations are not always linked to a viral reactivation. IgG avidity index is currently used in a few teams.[62]

Evidence of an active HCMV infection in a pregnant women does not allow us to conclude there is a fetal infection. However, if an abnormal fetal development is observed, it will be a strong argument in favor of maternal fetal transmission.

In children, the exceptional CID offers a picture of fetopathy (icteria, purpura, hepatomegaly, hypotrophy) along with cephalic signs (microcephaly, microophtalmy, cerebral periventricular calcifications, chorioretinitis). The age of pregnancy during maternal p.i. does not seem to interfere with the fetus' contamination risk.

The prognosis is very bad: 30% of CIDs are lethal, 60% lead to irreversible sequelae, while only 10% lead to normal development.

As for the asymptomatic infection of 1% of all neonates, it is responsible for heavy auditory long term disorders (10 to 15% variable deafness) and psychomotric retardation.

Neonatal infection diagnosis is seldom made without CID. Prenatal diagnosis, when ultrasonography is suspect, consists of amniocentesis from the 8th week or cordocentesis from the 17th week. Direct virus detection is made by standard and/or rapid cell culture [63] along with fetal IgM in cord blood and nonspecific signs like fetal thrombopenia.[64,65] A negative detection does not absolutely eliminate the diagnosis because the lag time between maternal infection and viral excretion in the fetus is not known.

In the newborn, virus isolation within the two first weeks of life, especially in urine, gives definitely the evidence of in utero acquired infection. Beyond this period, evidence of a HCMV viruria does not allow us any longer to differentiate a congenital from a perinatal infection.[66]

IgM is also assayed at birth, in particular in cord blood.

Two antiviral products are efficient against HCMV: foscarnet and ganciclovir. They are partially toxic, and they cannot change the acquired lesions of CID, thus they cannot be used systematically to prevent late handicaps linked to congenital asymptomatic infections.

Punctual trials are reported in the literature, from intraveinous infusion of ganciclovir in the umbilical vein to intravenous treatment of the neonate. Those treatments have at best reduced the viral titer without noticeable lessening of the symptoms. Ganciclovir is actually virostatic, not virocidic, and stops virus multiplication without eliminating it from the site of infection.[67] When ganciclovir is stopped viral excretion starts again.[68] The benefit of the treatment could be to allow the CNS to develop without bearing the consequences of too heavy a viral inoculum. Therapeutic abortion is very rarely indicated because of the lack of alarm signs of the mother.

### INTERVENTION STRATEGIES

Prevention of neonatal infection is almost impossible. Of course, seropositive donor blood must not be transfused to a seronegative pregnant woman. Day-nursery hygiene must be observed, since more than 10% of the children living in this community contract HCMV infection during their first year of life.

However, the most frequent means of infection among pregnant women can hardly be avoided: contamination of a seronegative mother by an elder child living in the community, which brings a prime infection risk during the current pregnancy.

Screening of exposed women is currently being evaluated in controlled studies. It is not systematicaly advised to date because of the inevitable maternal anxiety which cannot be compensated by rational solutions if she contracts a primary infection during her pregnancy.

The risk for the fetus can not be evaluated because the duration of the HCMV incubation in utero is not known, and the lag time between p.i. and amniocentesis is uncertain; also, should the risk of a second amniocentesis, if the first one is negative, or of cordocentesis be taken? If amniocentesis is positive, 50% of the fetuses are not infected.

So the only proposition should be therapeutic abortion or long term pediatric follow up until current studies can bring a consensus.[69]

The lack of an active vaccine against HCMV is stressed. Vaccine completion has become a priority of virologic fundamental research.[70]

### REFERENCES

1. Herold BC, WuDunn D, Soltys N, Spear PG: Glycoprotein C of herpes simplex virus type 1 plays a principal role in the adsorption of virus to cells and in infectivity. J Virol 65:1090–1098, 1991.
2. Shieh MT, Wu Dunn D, Montgomery RI, et al.: Cell surface receptors for herpes simplex virus are heparin sulfate proteoglycans. J Cell Biol 116:1273–1281, 1992.
3. Honess RW, Roizman B: Regulation of herpesvirus macromolecular synthesis. Cascade regulation of the synthesis of three groups of viral proteins. J Virol 14:8–19, 1974.
4. Kwong AD, Kruper JA, Frenkel N: Herpes simplex virus virion host shut off function. J Virol 62:912–921, 1988.
5. Liljelund P, Ingles CJ, Greenblatt J: Altered promoter binding of the TATA box-binding factor induced by the transcriptional activation domain of VP16 and suppressed by TFIIA. Mol Gen Genet 241:694–699, 1993.
6. Kieff ED, Bachenheimer SL, Roizman B: Size, composition and structure of the DNA of subtypes 1 and 2 herpes simplex virus. J Virol 8:125–129, 1971.
7. Hayward GS, Jacob RJ, Wadsworth SC, Roizman B: Anatomy of herpes simplex virus DNA: evidence for four populations of molecules that differ in the relative orientations of their long and short segments. Proc Natl Acad Sci USA 72:4243–4247, 1975.
8. Preston VG, Davison AJ, Marsden HS, et al.: Recombinants between herpes simplex virus types 1 and 2: analyses of genome structures and expression of immediate-early polypeptides. J Virol 28:499–517, 1978.
9. Braid S, Oury JF, Edinger C, et al.: Management/ Decision Making During Pregnancy. Eurogin Lower Genital Tract Infections and Neoplasia—3 rd International Congress 1997.
10. Berman PW, Dowbenco D, Lasky LA, Simonsen CC: Detection of antibodies to herpes simplex virus with a continuous cell line expressing cloned glycoprotein D. Science 222:524–527, 1983.
11. Nahmias AJ, Ee FK, Bechman-Nahmias S: Sero-epidemiological and sociological patterns of herpes simplex virus infection in the world. Scand J Infect Dis 69:19–36, 1990.
12. Boué A, Cabau A: Epidémiologie des infections par le cytomégalovirus. Nouv Pres Méd 7:3135–3139, 1978.
13. Maniez-Montreil M., Dupressoir MV., Huart JJ: Etude sérologique anti-cytomégalovirus (CMVH) d'une population de donneurs de sang. Nouv Rev Fr Hématol 27: 134, 1985.
14a. Stagno S, Pass RF, Dworsky ME, Alfrod CA: Maternal cytomegalovirus infection and perinatal transmission. Clin Obstet Gynecol 25:563–576, 1982.
14b. Stagno S, Pass RF, Dworsky ME, et al.: Congenital cytomegalovirus infection. The relative importance of primary and reccurent maternal infection. N Engl J Med 306:945–949, 1982.
15. Griffith PD, Babonian C: A prospective study of primary cytomegalovirus infection during pregnancy: final report. Br J Obstet Gynecol 91:307–315, 1994.
16. Ashraf SJ, Parande CM, Arya SC: Cytomegalovirus antibodies of patients in the Gizian Area of Saudi Arabia. J Infect Dis 152–1351, 1985.

17. Wang PS, Evans AS: Prevalence of antibodies to Epstein-Barr virus and cytomegalovirus in sera from a group of children in the People's Republic of China. J Infect Dis 153:150–152, 1986.
18. Sohn YM, Park KI, Lee C, Lee WY: Congenital cytomegalovirus infection in Korean population with very high prevalence of maternal immunity. J Korean Med Sci 7:47–51, 1992.
19. Ho: Rev Infect Dis 12 suppl 7:S 701, 1990.
20. Cabrera CV, Wholenberg C, Openshaw H: Herpes simpex virus DNA sequences in the CNS of latently infected mice. Nature 298:1068, 1978.
21. Ekwo E, Wong YW, Myers M: Asymptomatic cervicovaginal shedding of herpes simplex virus. Am J Obstet Gynecol 134:102, 1979
22. Zhang, et al.: Infect Dis 171:1002, 1995.
23. Toorkey, Carrigan: J Infect Dis 16024, 1989.
24. Reddhase, et al., J Exp Med 179:185, 1994.
25. Balachandran N, Bachetti S, Rawls WE: Protection against lethal challenge of BALB/c mice by passive transfer of monoclonal antibodies to five glycoproteins in herpes simplex virus type 2. Infect Immun 37:1132–1137, 1982.
26. Martin S, Courtney R, Fowler G, Rouse BT: Herpes simplex virus type 1 specific cytotoxic T lymphocytes recognize virus structure proteins. J Virol 62:2265, 1988.
27. Sullender WM, Miller JL , Yasukawa LL, et al: Humoral and cell-mediated immunity in neonates with herpes simplex virus infection. J Infect Dis 155:28–37, 1987.
28. Wronska, et al.: In: Michelson S & Plotkin SA (eds.): Multidisciplinary Approach to Understanding Cytomegalovirus Disease. Elsevier, p. 321, 1993.
29. Ridell, et al.: Proceedings of the 4th International Conference on HCMV, Elsevier Science Publishers, 1993.
30. Nakano, et al.: Cell Immunol 147:73, 1993.
31. Wahl SM, et al.: Immunol Today 10:258, 1989.
32. Alcami, et al.: J Virol 68:5730, 1994,
33. Nahmias AJ, Keyserling HL, Lee FK: Herpes simplex viruses 1 and 2. In: Evans AS (ed.): Viral Infections of Humans: Epidemiology and Control. New York: Plenum, pp. 393–417, 1989.
34. Brown ZA, Benedetti J, Ashley R, et al.: Neonatal herpes simplex virus infection in relation to asymptomatic maternal infection at the time of labor. N Engl J Med 324:1247–1252, 1991.
35. Hatherley LI, Hayes K, Jack I: Herpesvirus in an obsteric hospital. II. Asymptomatic virus excretion in staff members. Med J Aust 2:273–275, 1980.
36. Bogess KA, Watts DH, Hobson AC, et al.: Herpes simplex virus type 2 detection by culture and polymerase chain reaction and relationship to genital symptoms and cervical antibody status during the third trimester of pregnancy. Am J Obstet Gynecol 176:443–51, 1997.
37. Whiltley RJ, Corey L, Arvin A, et al.: Changing presentation of neonatal herpes simplex virus infection. J Infect Dis 158:109–116, 1988.
38. Whitley RJ, Arvin A, Prober C, et al.: Predictors of morbidity and mortality in neonates with herpes simplex virus infections. N Engl J Med 324:450–454, 1991.
39. Kimura H, Futamura M, Kito H, et al.: Detection of viral DNA in neonatal herpes simplex virus infections: frequent and prolonged presence in serum and cerebrospinal fluid. J Infect Dis 164:289–293, 1991.
40. Douglas J, Schmidt O, Corey L: Acquisition of neonatal HSV-1 infection from a partenal source contact. J Pediatr 103:908–910, 1983.
41. Braid S, Oury JF, Edinger C, et al.: Management/ Decision Making During Pregnancy: Eurogin 1997: Lower Genital Tract Infections and Neoplasia—3rd International Congress.
42. Ahlfors K, Forsgren M, Ivarsson SA, et al.: Congenital cytomegalovirus infection: On the relation between type and time of maternal infection and infant's syndrome Scan J Infect Dis 15:129–138, 1983.
43. Boppana SB, Pass RF, Britt WJ, et al.: Symptomatic congenital infection: neonatal morbidity and mortality. Pediatr Infect Dis J 11:93–99, 1992.
44. Saltzman RL, Quirk MR, Jordan MC: Disseminated cytomegalovirus infection. Molecular analysis of virus and leukocyte interaction in viremia. J Clin Invest 81:75–81, 1988.
45. Goff E, Griffith BP, Booss J: Delayed amplification of cytomegalovirus infection in the placenta and maternal tissues during late gestation. Am J Obstet Gynecol 156: 1265–1270, 1987.
46. Van der Bij W, Toresma R, Van Son WJ, et al.: The rapid immunodiagnosis of active cytomegalovirus infection by monoclonal antibody staining of blood leukocytes. J Med Virol 25:179–788, 1988.
47. Cabau N, Labadie MD, Vesin C, et al.: Seroepidemiologie of cytomegalovirus infections during the first years of life in urban communities. Arch Dis Child 54: 286–290, 1979.
48. Audra Ph, Aymard M, Gibert R: Cytomégalovirus et grossesse. Méd Mal Infect Numéro spécial: 44–47, 1988.
49. Stagno S, Pass RF, Cloud G, et al.: Primary cytomegalovirus infection in pregnancy. Incidence transmission to fetus and clinical outcome. JAMA. 256:1904–1908, 1986.
50. Stern H, Tucker SM: Prospective study of cytomegalovirus infection in pregnancy. Br Med J 2:268–270, 1973.
51. Griffith PD, Stagno S, Pass RF, et al.: Infection with cytomegalovirus during pregnancy: specific IgM antibodies as a marker of recent primary infection. J Infect Dis 145:647–653, 1982.
52. Vial P, Torres J, Stagno S: Serological screening for cytomegalovirus rubella virus, herpes simplex virus, hepatitis B virus, and Toxoplasma gondii in two populations of pregnant women in Chili. Bol Sanit Panam 99:528–538, 1985.
53. Kamada M, Komori A, Chiba A, Nakao T: A progressive study of congenital cytomegalovirus in Japan. J Infect Dis 15:227–232, 1983.
54. Chandler S, Hansfield HH, Mc Dougall JK: Isolation of multiple strains of cytomegalovirus from women attending a clinic for sexually transmitted diseases. J Infect Dis 155:655–660, 1987.
55. Spector SA: Systemic infection with multiple strains of cytomegalovirus assassed by restriction enzyme digestion analysis. Pediatr Res 16:1383, 1982.

56. Nankeris GA, Kumar ML, Cox E, Gold E: A prospective study of maternal cytomegalovirus infection and its effect on the fetus. Am J Obstet Gynecol 149:435–440, 1984.
57. Dworski M, Yow M, Stagno S, Pass RF, Alford C: Cytomegalovirus infection of breast milk and transmission in infancy. Pediatrics 72:295–299, 1983.
58. Amin H, Jadavji T, Saave R, Gill J: Use of ganciclovir in the treatment of acquired cytomegalovirus disease in preterm infant. Can J Infect Dis 1:28–30, 1990.
59. Kumar ML, Nankeris GA, Cooper AR, Gold E: Postnatally acquired cytomegalovirus infections in infants of CMV–excreting mothers. J Pediatr 104:669–673, 1984.

60a. Fowler KB, Stagno S, Pass RF, et al.:The outcome of congenital cytomegalovirus infection in relation to maternal antibody status. N Engl J Med 326:663–667, 1992.

60b. Fowler KB, Stagno S, Pass RF: Congenital cytomegalovirus infection and maternal antibody status. N Engl J Med 327:496, 1992.

61. Revello MG, Percivalle E, Baldanti F, et al.: Prenatal treatment of congenital human cytomegalovirus infection by fetal intravascular administration of ganciclovir. Clin Diag. Virol 289:1–5, 1993.
62. Dussaix E, Chantot S, Harzic M, Grangeot-Keros L: CMV-IgG avidity and CMV-IgM concentration in both immune-compromised and immunocompetent patients. Pathol Biol 44:405–410, 1996.
63. Mazeron MC, Jahn G, Platcher B: Monoclonal antibody E-13 (McAb 810) to human cytomegalovirus recognizes an antigen encoded by exon 2 of the major immediate-early gene. J Gen Virol 73:2699–2703, 1992.
64. Cordovi-Voulgaropoulos L: Diagnostic de l'infection congénitale à CMVH. A propos d'une observation. Thèse pour le Doctorat en Médecine, Faculté de Médecine Saint-Antoine, Paris, 1987.
65. Daffos F: Diagnostic des infections virales chez le foetus. In: Azoulay M, Delfraissy JF (eds.): Virus et Grossesse. Les Editions Inserm, 1992.
66. Sawer MH, Edwards DK, Spector SA: Cytomegalovirus infection and bronchopulmonary dysplasia in premature infants. Am J Dis Child 151:303–305, 1987
67. Fan-Havard P, Nahata MC, Brady T: Ganciclovir: a review of pharmacology, therapeutic efficacy and potential use for treatment of congenital cytomegalovirus infections. J Clin Pharm Therap 14:329–340, 1989.
68. Demmler GJ: Summary of a workshop on surveillance for congenital cytomegalovirus disease. Rev Infect Dis 13:315–329, 1991.
69. Pass RF: Is there a role for prenatal diagnosis of congenital cytomegalovirus infection Pediatr Infect Dis 11: 608–609, 1992.
70. Porath A, McNutt RA, Smiley LM, Weigle KA: Effectiveness and cost benefit of a proposed live cytomegalovirus vaccine in the prevention of congenital disease. Rev Infect Dis 12:31–40, 1990.
